# Nanotechnologies in Glycoproteomics

**DOI:** 10.1186/1559-0275-11-21

**Published:** 2014-05-13

**Authors:** Hu Zhao, Yaojun Li, Ye Hu

**Affiliations:** 1 Department of Nanomedicine, Houston Methodist Research Institute, Houston, TX 77030, USA; 2 Department of Cell and Developmental Biology, Weill Cornell Medical College, New York City, NY 10021, USA

## Abstract

Protein glycosylation, as an important post-translational modification, is implicated in a number of ailments. Applying proteomic approaches, including mass spectrometry (MS) analyses that have played a significant role in biomarker detection and early diagnosis of diseases, to the study of glycoproteins or glycopeptides will facilitate a deeper understanding of many physiological functions and biological pathways involved in cancer, inflammatory and degenerative diseases. The abundance of glycopeptides and their ionization potential are relatively lower compared to those of non-glycopeptides; therefore, sample enrichment is necessary for glycopeptides prior to MS analysis. The application of nanotechnology in the past decade has been rapidly penetrating into many diverse scientific research disciplines. Particularly in what we now refer to as the “glycoproteomics area”, nanotechnologies have enabled enhanced sensitivity and specificity of glycopeptide detection in complex biological fluids, which are critical for disease diagnosis and monitoring. In this review, we highlight some recent studies that combine the capabilities of specific nanotechnologies with the comprehensive features of glycoproteomics. In particular, we focus on the ways in which nanotechnology has facilitated the detection of glycopeptides in complex biological samples and enhanced their characterization by MS, in terms of intensity and resolution. These studies reveal an increasingly important role for nanotechnology in helping to overcome certain technical challenges in biomarker discovery, in general, and glycoproteomics research, in particular.

## Introduction

Protein glycosylation is one of the most common post-translational modifications (PTMs) in living cells [1,2]. Glycoproteins play a vital role in biological processes including cell adhesion, receptor activation, and signal transduction [3-5]. As such, altered or erroneous glycosylation is often associated with inflammatory diseases, neurodegenerative disorders, and even certain cancers [6,7]. For example, several investigators have observed that the abnormal expression of different glycosylated proteins were implicated in disease and that changes in the level of glycoprotein could be used as hallmarks for disease diagnosis, including the carbohydrate antigen CA-19-9 for colon cancer [8], the prostate-specific antigen (PSA) for prostate cancer [9], α-fetoprotein for liver cancer [10], and β-human chorionic gonadotropin for germ cell tumors [11]. Furthermore, many accessible, membrane-bound or extracellular proteins are glycosylated and could be exploited for therapeutic intent, such as the Her2 receptor for breast cancer therapy [12].

**Figure 1:**
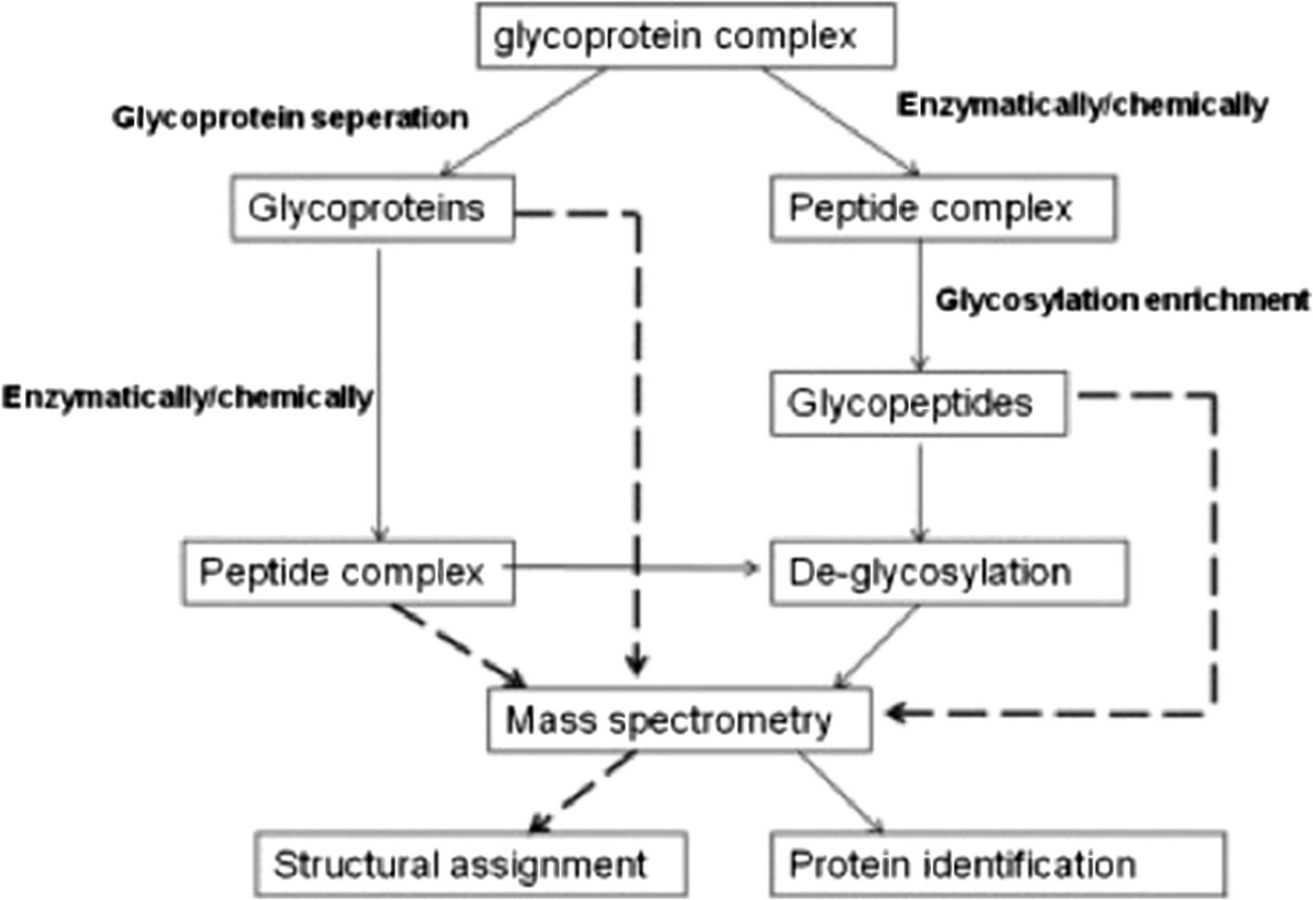
**Typical workflow for glycoproteomics analyses **[1]**.**

Thus far, analytical methods of identifying (sometimes subtle) changes in disease-relevant glycoproteins can be divided into two main groups: glycoprotein- or glycopeptide-based analysis. We illustrate the workflow of these two approaches in Figure 1[1]. In the first (glycoprotein-based) approach, glycoproteins are enriched by different separation methods, such as size exclusion, ion exchange, affinity chromatography, chemical immobilization, and other methods. To identify the protein part of a glycoprotein is much easier than to identify the glycan part of the same glycoprotein. In the latter (glycopeptide-based) strategy, the glycoproteins initially undergo enzymatic or chemical degradation, and the resulting glycopeptides can be enriched by several methods, such as lectin-affinity chromatography [13-15], boronic acid-based approach [16,17], hydrazide chemistry [18-20], or solid-phase extraction using hydrophilic interactions [21]. The enriched glycopeptides are then deglycosylated and quantified via MS analysis. Consequently, the sequences of various glycopeptides and their specific glycosylation sites can be easily identified. With the advent of ever more sophisticated MS modalities, we can now delve even deeper in the realm of glycoproteomics research. Still, challenges remain for profiling glycopeptides in complex samples (e.g., serum or blood) using current MS-based techniques, because the proportion of glycopeptides in total solution is quite small, and their signals in MS profiles are often obscured by those of non-glycosylated peptides [22]. Therefore, enhancing the specificity and sensitivity of glycopeptide enrichment and detection in complex samples remains a very real challenge.

**Figure 2:**
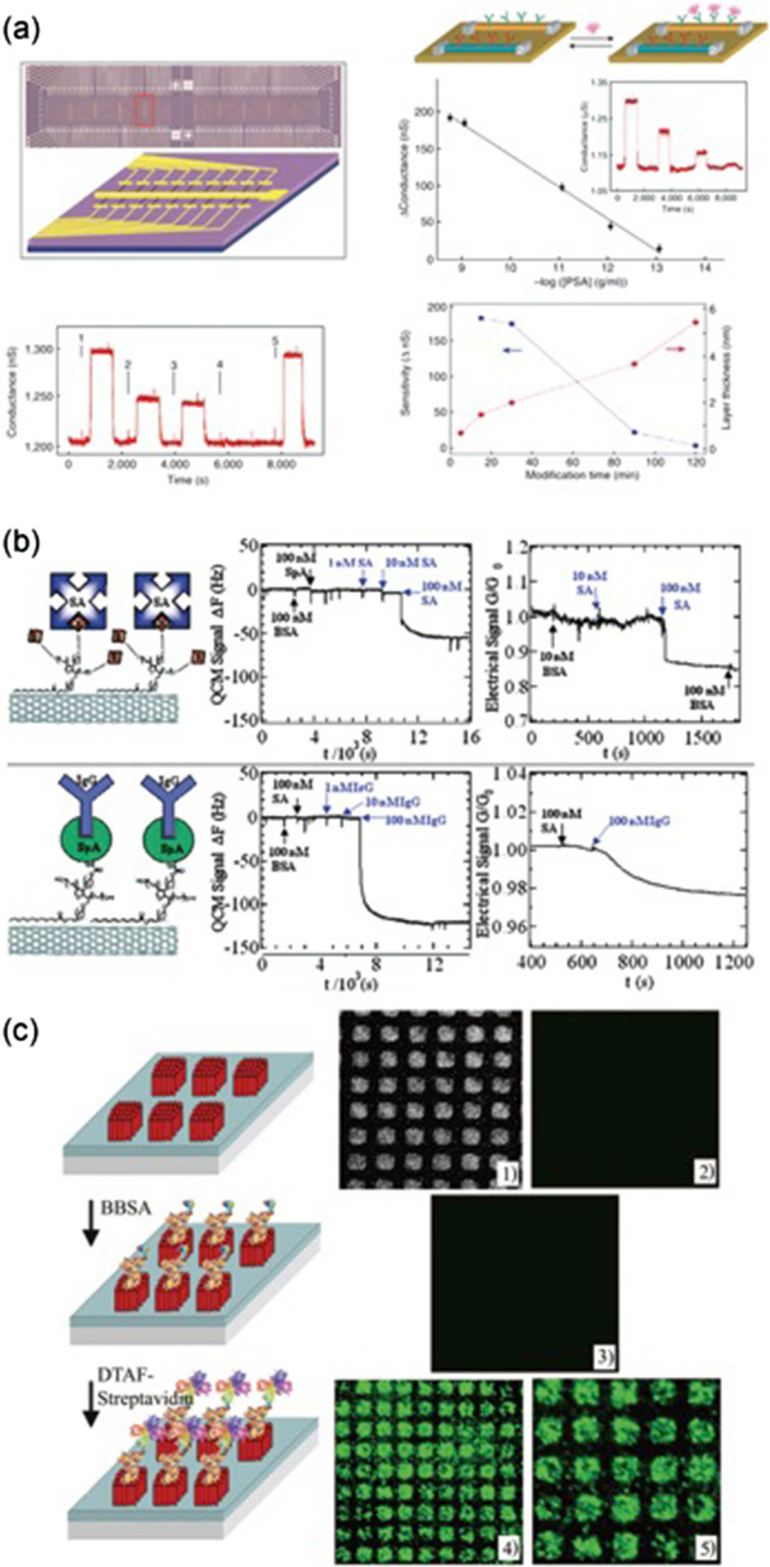
**Examples of nano-dimensioned biosensors. (a)** SiNW FET sensors are used to detect prostate-specific antigen (PSA) [29]; **(b)** Real-time Quartz Crystal Microbalance (QCM) and electrical sensing of model proteins [30]; and **(c)** Enhanced fluorescence detection carried out on various biological systems coupled to ZnO nanorod platforms. Scanning electron microscopy (panel 1) and fluorescence (panels 2 through 5) images of biotinylated bovine serum albumin (bBSA) and fluorescein derivativeconjugated streptavidin (DTAF-strept) on ZnO nanorods [31].

In the past decade, we have witnessed an enormous growth in the application of nanotechnology to solve biological and biomedical problems. Due to some of these (nano) technological advances, we can now identify and analyze biomolecules at a speed and resolution never seen before. Compared to conventional macro-scale materials, nano materials, such as nanoparticles, nanowires, nanotubes, nanorods, and thin films with meso-scale pores, have unique transport properties (i.e., more efficient electron transport), better optical excitation and high detection efficiency. Nanomaterials also impart to their fabricated platforms higher surface-to-volume ratio with reduced dimensionality, which are key features that often enhance the physical and chemical properties of materials. In addition, materials with lower dimensionality are conducive to extremely high unit-density integration on arrays and lab-on-a-chip platforms for point-of-care devices for high-throughput biodetection. Miniaturized devices composed of nanomaterials have the advantage of low cost, good portability, and potential use in minimally invasive instrumentation [23]. In Figure 2, we present some examples of nanomaterial-enabled platforms for biomolecule detection, such as silicon nanowire field-effect transistor (SiNW-FET) sensors for detecting prostate-specific antigen (PSA), Real-time Quartz Crystal Microbalance (QCM) for detecting serum proteins and ZnO nanorod platforms for detecting protein interaction. We and others have developed a series of mesoporous silica thin film chips with a variety of nanotextures that can selectively enrich for low molecular weight peptides from complex biological fluids, as part of a biomarker discovery platform [24-28].

Glycoproteins are not as amenable to isolation and identification due confounding factors such as complex glycan structures, glycan isomers, and amino acid residues attached to the oligosaccharide chains. The most commonly employed technique for the analysis of protein glycosylation (glycosylation site identification, glycan structure determination, site occupancy, and glycan isoform distribution) is MS, albeit with still a few existing technical challenges [32]. In this review, we will highlight some of the platforms that have been developed for the enrichment of glycopeptides from complex samples, and introduce emerging combination platforms that couple the sensitivity and accuracy of MS to the exquisite selectivity of nano-platforms.

## Different nano-platforms used to enrich glycopeptides

A nanomaterial is a natural, incidental or manufactured material containing particles in an unbound, aggregate or agglomerate state, where one or more external dimensions is in the size range 1–100 nanometers. Due to their smaller size, nanomaterials possess incredibly larger surface areas than bulky materials. This feature provides more activated sites for glycopeptide enrichment and unique optical properties, in the context of rapid and sensitive detection. Perhaps the most attractive characteristic of nanomaterials is the degree to which their size, shape and morphology can be precisely controlled to enhance the optical, electronic, or magnetic capabilities typically desired in certain applications. Several nano-platforms exist for the isolation of glycopeptides in complex samples with improved sensitivity and specificity, and we discuss them in more details in the following Subsections.

### Nanoparticles for glycopeptide enrichment

Nanoparticles are arguably the most widely used nanomaterial to assemble chips for biomolecule enrichment because: 1) nanoparticles can be synthesized with various size distributions, and morphologies; 2) nanoparticles can be modified and diversified by numerous reagents for specific uses; and 3) they exhibit unique optical and electrochemical features. When attempting to enrich glycopeptides in complex samples, researchers often adopt core-shell structured nanoparticles with a magnetic core within a thin layer of compatible material. The magnetic core, which provides a strong magnetic force, would be simply attracted by magnets and the outer layer would be easily modified by various organic or inorganic reagents to recognize the biomarker of interest.

Using a version of the above approach, Zhou et al. fabricated aminophenyl boronic acid-functionalized magnetic nanoparticles and used them to selectively capture glycopeptides and glycoproteins from mixtures containing non-glycomoleculars [33]. Other groups have also applied versions of surface-modified nanoparticles for glycoprotein selection. For example, 3-aminopropyltriethoxysilane (APTES) is frequently used as a surface-modifying reagent for silica nanoparticles, whereby the amino group imparts a positive charge. Zhang Y. and coworkers designed a one-step salinization reaction to assemble APTES onto the nanoparticle surface in order to capture aldehyde groups on oxidized glycopeptides. These nanoparticles greatly reduced the coupling time from 12–16 hours to just 4 hours without sacrificing enrichment efficiency, compared to traditional solid-phase extraction methods based on hydrazide resins [34]. Zhang L. et al. developed a novel composite material with a core-satellite structure. These are gold nanoparticles, functionalized with boronic acid, and then anchored on the surface of the silica-coated magnetic core. Using this strategy, the investigators were able to recover 85.9% of glycopeptides and 71.6% of glycoproteins after enrichment. The composite nanoparticles had an adsorption capacity of more than 79 mg of glycoproteins per gram of the material. The investigators used these new composite nanoparticles to enrich glycosylated proteins from human colorectal cancer tissues for subsequent identification of N-glycosylation sites. They were able to map 194 unique glycosylation sites, of which 165 sites (85.1%) were newly identified, to 155 different glycoproteins [35].

Polysaccharides can also be used as a surface-modifying component for glycopeptide enrichment. Xiong et al. reported a layer-by-layer approach to synthesize magnetic nanoparticles (MNPs) that are coated with multilayered polysaccharide shells, and then used these hydrophilic materials for selective enrichment of glycopeptides from biological samples. These investigators identified 605 unique N-glycosylation sites in 616 distinct glycopeptides, corresponding to 350 glycosylated proteins in 20 μg mouse liver protein sample. Their results indicated that exploiting specific sugar-sugar interactions shows promise as a design strategy for characterizing protein glycosylation [36].

Rather than using boronic acid as a surface-modifying reagent for nanoparticles, Tran et al. developed a new kind of gold nanoparticles, functionalized with ultra-small hydrazide groups, with a core diameter of 1.2 nm. The assembly process involves an oxidation step and covalent coupling, whereby the carbohydrate moiety of the glycoproteins are oxidized into aldehydes by periodate and the oxidized glycoproteins are covalently coupled to hydrazide resin. No glycoproteins are removed by washing. They successfully utilized these nanoparticles to isolate 90% of the glycopeptides from complex biological samples. The nanoparticle stability in biological solution, unique solubility, and large capacity for peptide capturing provide great application potential in glycoproteomics studies [37].

Isolating glycopeptides via their lectin moiety is another common approach, and commercialized solutions are now available in the market. Tsutsumi and colleagues conjugated gold nanoparticles (GNPs) with monosaccharide-modified peptides as optical probes for lectin detection. The aggregation of the glycopeptide-modified GNPs with concanavalin A (ConA) causes an absorption shift from 534 nm to 620 nm, an apparent color change that can be detected by the naked eye [38].

Ligand-modified nanoparticles have been proven to be an excellent platform for glycopeptide enrichment, but ligand-free nanoparticles are emerging as good candidates for glycoproteins or glycopeptides isolation. A research group in Fudan University developed ligand-free silver nanoparticles coated with magnetic nanoarchitecture for selective enrichment of glycopeptides by taking advantage of the reversible interaction of glycans with silver nanoparticles. The investigators could easily and quickly (only 1 min incubation time) extract glycopeptides at a low molar ratio of glycopeptides: non-glycopeptides (1:100) using these silver-nanoparticle magnetic beads. Moreover, they mapped 127 unique glycopeptides mapped to 51 different glycoproteins from a very small sample volume (only 1 μL rat serum) [39].

Particle-based materials are the most widely adopted for isolating and separating glycosylated peptides due to their nano dimensionality, tightly controlled size, and unique optical and electrochemical properties. They will likely continue to play a prominent role in glyco-proteomics for biomarker detection and early diagnosis of diseases.

### Mesoporous materials for glycopeptide enrichment

Several classes of porous materials exist and group according to size. The International Union of Pure and Applied Chemistry (IUPAC) clarifies [40], microporous materials and macroporous materials as having pore diameters less than 2 nm and greater than 50 nm, respectively; mesoporous materials place in the middle. Developed since the 1970’s, mesoporous silica materials are, at present, widely used in drug delivery, imaging, biosensors, for example, owing to their regular pore arrangement, simple preparatory methods, and low cost. Under the influence of strong capillary forces, biomolecules entering the mesoporous pores encounter more activation sites for binding, resulting in higher separation efficiency. Many glycoproteomics studies have been conducted using mesoporous materials for glycopeptide isolation and separation.

**Figure 3:**
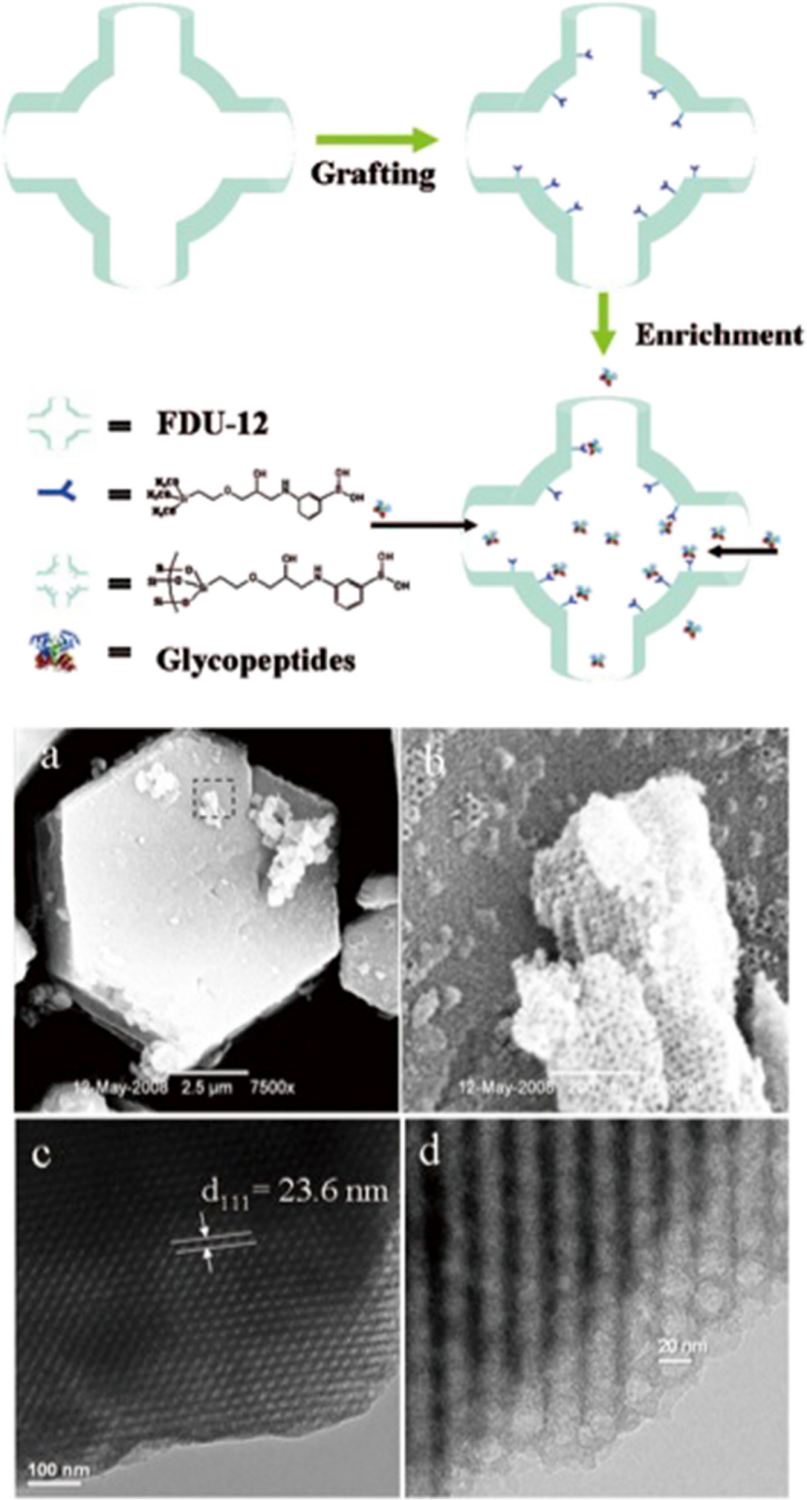
**A schematic of glycopeptide enrichment from dilute solutions using mesoporous silica material modified with boronic acid.** The pores act as a nanoreactor for the specific binding of di-boronic acid groups to glycopeptides (top half of the figure). FE-SEM images of boronic acid-modified mesoporous silica material at low magnification **(a)** and the high-resolution image of the squared area in part a; and **(b)** TEM images along the vertical direction of the mesoporous materials **(c,d)**[41].

Xu and colleagues first introduced mesoporous silica materials functionalized with boronic acid for the detection of glycosylated peptides. In Figure 3, we illustrate the basic workflow of this approach. The field emission electron scanning microscope images of the prepared products show a pattern of regular honeycomb-like hexagonal-patterned pores. Glycopeptides in solution bound with high affinity to the groups attached within the pores, markedly improving the solute detection limit [41]. Similarly, Liu et al. [22] and Zhang H. et al. [42] applied mesoporous silica functionalized with aminophenylboronic acid (APB) to analyze glycopeptides in standard protein solutions and rat serum, respectively.

**Figure 4:**
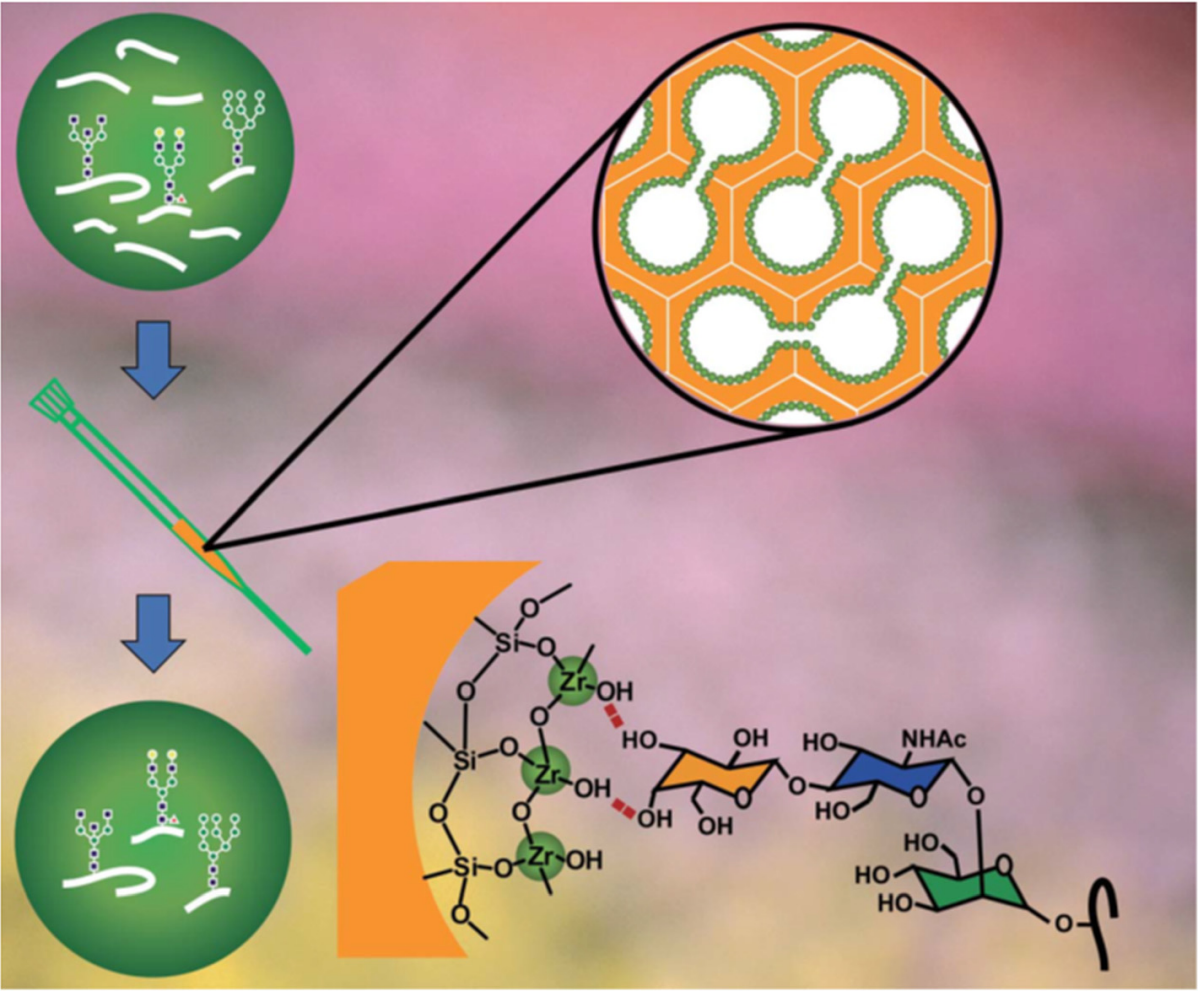
**A schematic representation of glycopeptide isolation using mesoporous silica microspheres coated with zirconia**[43]**.**

Other than boronic acid, transition metal oxides can be coupled to mesoporous silica, as demonstrated by Wan and coworkers. These investigators coated mesoporous silica microspheres in a layer of zirconium dioxide to create a platform for hydrophilic interaction liquid chromatography (HILIC) solid phase extraction (SPE) (depicted in Figure 4). HILIC has broad glycan specificity, good reproducibility, and compatibility with MS analysis. ZrO_2_ species were highly dispersed on the surface of meso-structured cellular foam. Hydrogen bonding between the oxygen atoms of ZrO_2_ and the hydrogen atoms of glycans forms a relatively strong interaction, thus isolating the glycopeptides from complex samples. Since ZrO_2_ can also attract phosphopeptides, the investigators treated digested mixtures of the phosphoprotein, α-casein, and IgG with ZrO_2_/MPS HILIC SPE materials. The results demonstrated that glycopeptides could be effectively enriched with interference from Zirconia layer coated silica surface [43].

Oxidized mesoporous carbon is another very useful material in glycoproteomics research because of its extraordinary hydrophilicity. Hence, oligosaccharides on N-linked glycans interact with the carbon functional groups, enabling glycopeptide separation prior to profiling by MS. Using this approach, Qin et al. enriched N-linked glycans using oxidized and ordered mesoporous carbon materials of CMK-3 before matrix-assisted laser desorption/ionization time-of-flight (MALDI-TOF) MS. Twenty four N-linked glycans derived from standard glycoproteins could be detected with high signal-to-noise (S/N) ratios and peak intensities. Thirty-two N-linked glycans were profiled in complex samples, five (4 core-fucosylated glycans) of which exhibited distinct patterns in liver cancer compared to healthy samples. Their results support the notion that precisely-sized mesoporous carbon materials could play a prominent role in clinical glycoproteomics [44].

With such broad applicability and adaptability, we expect the use of mesoporous materials to continue leaving an impact on the glycoproteomics landscape. The large specific surface area, surface modification potential, size exclusion toward large molecular weight biomolecules, and support strong capillary force in nano scale are all characteristics that lend well to their wide-spread use.

### Other nano platforms for glycopeptide enrichment

**Figure 5:**
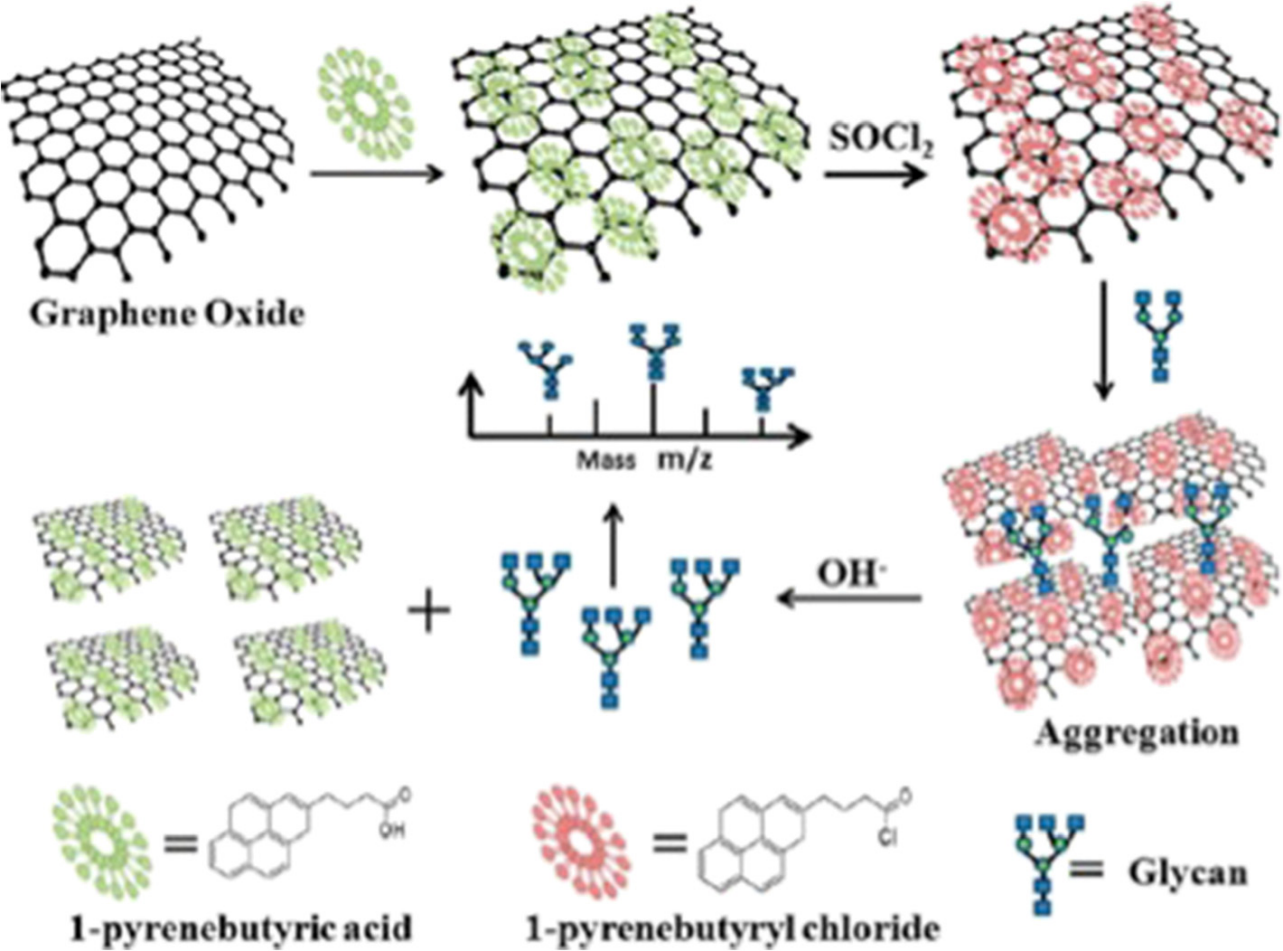
**Using functionalized graphene for glycan separation **[46]**.**

Two-dimensional (2D) crystalline materials have recently been identified and analyzed for use in glycopeptide enrichment [45]. Graphene, consisting of a single atomic layer of carbon, is the first applied in this new class of materials. A number of unique properties make it interesting and suitable for both fundamental research and future applications. Due to its large specific area and ultra-strong absorbability, surface-functionalized graphene or graphene oxide is now used for glycan enrichment. Zhang et al. recently introduced a rapid, highly efficient, and visual approach for glycan enrichment using 1-pyrenebutyryl chloride functionalized graphene oxide [46], illustrated in Figure 5. First, graphene was functionalized by 1-pyrenebutyric acid and following 1-pyrenebutyryl chloride treatment. Reversible covalent bonding occurs between the hydroxyl groups of the glycans and the acyl chloride groups on graphene oxide via the π-π stacking of 1-pyrenebutyryl chloride. Enrichment efficiency was much improved by the large specific surface area and heavy functionalization of active 1-pyrenebutyryl chloride. When the investigators tested this material in several sample processing applications, MS signal intensity, signal-to-noise ratio, and the number of glycoforms identified on standard oligosaccharides or the N-glycans that were released from glycoproteins increased remarkably.

Carbon nanotubes (CNTs) are allotropes of carbon exhibiting a cylindrical nanostructure. These cylindrical carbon molecules have unusual properties that could be, and is currently, exploited by the electronics industry, optics industry, and other fields of materials science and technology. Zhang X. and colleagues constructed a novel lectin-based electrochemical biosensor of functionalized, multi-walled CNTs to capture glycans from living cells [47]. They fabricated the biosensor by adsorbing poly(diallyldimethylammonium chloride) (PDDA)-functionalized CNTs (PDCNTs) onto a glassy carbon electrode (GCE), followed by glutathione (GSH)-protected gold nanoparticles (AuNPs) adsorption. The resulting material becomes an effective platform for lectin immobilization, with high stability and bioactivity. The investigators also synthesized a thiol-derivatized carbohydrate (thiomannosyl dimer) to construct (CNT/thionine/Au–S–mannose) biocomposites, which used CNTs as carriers to load enormous amounts of thionine (to generate an electrochemical signal) and AuNPs (to anchor the thiomannosyl dimer).

The two applications for functionalized CNTs, whether as a biosensing platform and biocomposite, allow enhanced glycan signal detection. The former (electrochemical biosensor) has demonstrated good analytical performance – high sensitivity, selectivity and rapid response – for the analysis of mannose in human lung cancer cells. This approach could be adapted for other indications by broadening the selection moiety (e.g., lectins) for biosensor development.

## Conclusion and perspective

Investigators have long recognized the significance of protein glycosylation in physiological processes and disease, as evidenced by the number of related publications that continue to accumulate. Furthermore, glycosylated proteins are increasing identified as biomarkers for early disease detection of diseases via proteomics methods. Although recent advances in mass spectrometry have made large-scale identification of proteins feasible, it is still very challenging to analyze protein glycosylation in complex samples owing to the fact that glycopeptides often constitute a minor portion of the total peptide mixture, whose signal intensities are often lower compared to non-glycosylated peptides and are suppressed in the presence of non-glycopeptides. Nanotechnology has undoubtedly contributed to the realization of glycoprotein/glycopeptide enrichment methods, particularly from enrichment from complex samples in preparation for MS characterization. The use of nanomaterials with various morphologies and characteristics (nanoparticles, mesoporous materials, nanosheets, nanotubes, etc.) has enabled enhanced sensitivity and specificity of glycopeptides isolation.

Challenges do remain in detecting post-translationally modified proteins/peptides, especially circulating glycopeptides. Characterizing glycosylated peptides is also difficult due to unique electrochemical properties, optical properties, and energy brand structure. By applying nano-scaled materials to tunable platforms, scientists have greatly enhanced the capabilities of traditional MS-based techniques, by increasing mass resolution, mass range, mass intensities, to name a few. We believe this significant progress blazes a trail for future efforts (and accomplishments) in the combined areas of nano-engineering, MS and glycoproteomics.

## Competing interests

The authors declare that they have no competing interests.

## Author’s contributions

HZ, YL and YH participated the drafting of the manuscript. All of the authors read and approved the final manuscript.
